# Flipping the expert: faculty educator sensemaking during transition to an active learning-based curriculum

**DOI:** 10.1186/s12909-024-05039-4

**Published:** 2024-01-23

**Authors:** Joanna Veazey Brooks, Dorothy Hughes

**Affiliations:** 1 Departments of Population Health and Palliative Medicine, University of Kansas School of Medicine Kansas City, 3901 Rainbow Blvd, MS 3044, Kansas City, KS 66160 USA; 2 Departments of Population Health and Surgery, University of Kansas School of Medicine, Salina, KS USA

**Keywords:** Curriculum, Undergraduate medical education, Teaching, Problem-based learning

## Abstract

**Purpose:**

Curricular change is becoming a standard feature of medical schools as they respond to learners’ evolving needs. Implementing change is not always straightforward, however, especially when it directly shifts the expected roles of faculty educators. The authors investigated how faculty educators navigated a significant transition to the Active, Competency-Based, and Excellence-Driven (ACE) curriculum at one state medical school.

**Method:**

The authors employed a qualitative descriptive design and conducted thematic analysis. From June 2018 to January 2019, the authors conducted individual, in-depth interviews with faculty educators and administrators involved in first-year medical student education. Data were analyzed inductively to identify the sensemaking process for faculty.

**Results:**

Twenty-one faculty educators participated in interviews averaging 58 min. Four phases were identified among educators as they moved through the change: (1) Making Sense of the Change; (2) Grieving the Lecturer Educator Role; (3) Risking an Active Learning Educator Role; and (4) Identifying the Rewards of Active Learning-based Teaching.

**Conclusion:**

Faculty buy-in is an essential component of successful curricular change implementation. While most faculty in this study reported eventual enjoyment from the new interactional teaching that fostered critical thinking, navigating the change was not always smooth. This study suggests faculty development around curricular change should be tailored to address the varying faculty concerns relevant to the four phases that were identified. Effective and optimal faculty support during large-scale curricular change must take into account not just new skills but also the grief and risk faculty may experience as their roles shift.

## Background

Despite general consensus about the continued need for curricular change in medical schools and the broad goals of such change [1–3], the process of actually accomplishing effective curricular change is not fully understood. In fact, debate about the implementation of curricular change —including its scope and content—is common and long-standing. Discussions around large curricular transformation cite the culture and political environment around the change as central to the process [4].

While student outcomes and perceptions are monitored closely during curricular change, faculty educators’ experiences are also central to the process but not similarly monitored [5–7]. The degree of faculty support or resistance is a key driver of the local cultural response to curricular change and its implementation’s success [4, 8]. In fact, one school identified eager support from faculty as a key facilitator and simultaneously identified faculty resistance as a key barrier to their new undergraduate medical education (UME) curriculum [9]. Even for faculty who desire to be supportive of curricular change, adapting can be difficult, especially when being an educator is one of many roles competing for attention and time [10, 11]. Further, some curricular change, like the one examined in our study, required not just a shift in content but also a fundamental change in educator role.

While a number of studies on professional identity formation have examined the process by which clinicians [12–15] or scientists [16, 17] take on an educator identity in medical education, the process of shifting from one educator type to another within medical education (e.g. lecture-based educator to active-learning educator) is unclear. Existing models of professional identity formation highlight roles, agency, and context as three key forces influencing identity [10, 18]. Our study provides a unique opportunity to study the change in roles and agency when the context of educating (i.e. the curriculum) drastically changes. Prior work on teaching-centered and learning-centered orientations have shown that a change from one orientation to the other is profound, requiring faculty to change their central beliefs about educating [19, 20]. Of note, scholars have shown that shifts between these do not “automatically take place,” even when the curriculum changes [20, 21].

Given the central support of faculty for successful curricular change coupled with existing research showing that navigating such change is challenging, we sought to investigate and understand the sensemaking process faculty educators used to navigate a substantial curricular change from the former legacy curriculum [22] to an active learning curriculum. By sensemaking, we refer to the social process of understanding the shifting environment and one’s place in it [23, 24]. With greater understanding of faculty sensemaking around curricular change, medical educators– and likely health professions educators more broadly– may be better able to anticipate faculty needs, build buy-in, and thus weather future change more smoothly.

## Methods

### Study setting

Our medical school implemented a comprehensive curricular change in 2017. The new curriculum is referred to as ACE for Active-learning, Competency-based and Excellence-driven education [25] and replaced our legacy curriculum [26, 27]. This curricular change was reported by Novak et al. (2019) to be a “radical redesign,” one of only two schools they examined whose curricular change was given that categorization [28].

### Study design

The authors designed a qualitative descriptive study using semi-structured interviews for data collection and conducting a thematic analysis using grounded theory elements including memoing and constant comparison [29, 30]. Using purposive sampling, we recruited faculty educators and administrators on all three medical school campuses (main, large regional, and small regional) who had extensive involvement with first-year medical students (M1s) during the first year of ACE implementation and at least one year prior. ACE was implemented sequentially over four years, starting with the M1s and engaging all students by year four. Our recruitment strategy captured individuals who had experience teaching in the legacy curriculum and in ACE.

### Data collection and analysis

From June 2018 to January 2019, the authors collected data by conducting one-on-one, in-depth interviews with faculty educators and administrators using a semi-structured interview guide based on concepts in the relevant literature and consultation with staff in the Office of Medical Education. All participants signed a written informed consent prior to the interview, and the study was approved by our Institutional Review Board. Interviews were audio-recorded and professionally transcribed verbatim.

Authors conducted the interviews together when possible and independently when scheduling required it. During the study period, one author was faculty in a non-clinical department in the School of Medicine; all participants were School of Medicine faculty across various departments. However, the author was not teaching M1 students, nor did they have any shared administrative responsibilities with participants. The other author was a PhD student during the study period and had limited previous exposure to any study participants. The distance between the authors and the subject matter translated to a significant degree of objectivity and few preconceived notions. In addition, memoing and constant comparison [31], both techniques in the grounded theory tradition, were used throughout the data collection period and additionally facilitated reflexivity.

To analyze the data, the authors collaboratively developed a codebook with codes and code definitions based on initial coding of five interviews, then refined the codebook iteratively throughout the coding process. Remaining interviews were divided between the two authors, one assigned as primary coder and the other a secondary reviewer. The authors met as needed to discuss any questions or differences in coding. Our analytic approach employed thematic analysis which allowed for inductive identification of descriptive themes and findings related to the sensemaking process experienced by faculty [32]. Analysis was managed with NVivo QSR International Software. Illustrative quotes are used throughout; participants are identified by randomly assigned numbers in brackets.

## Results

Twenty-one faculty educators and administrators participated in individual interviews; mean interview length was 58 min. Table 1 reports participants’ demographic characteristics. Four key phases describe the process educators experienced during the change to active teaching and its impact on their educator identities: (1) Making Sense of the Change; (2) Grieving the Lecturer Educator Role; (3) Risking an Active-Learning Educator Role; and (4) Identifying the Rewards of Active Learning-Based Teaching.

**Table 1:** Characteristics of participants (n = 21)

Demographic characteristics	No. (%)
**Gender**	
Male	15 (71.4)
Female	6 (28.6)
**Degree**	
MD/DO	13(61.9)
PhD	8 (38.1)
**Year PhD or MD obtained**	
Prior to 1980	3 (14.3)
1980–1989	7 (33.3)
1990–1999	6 (28.6)
2000 or after	5 (23.8)
**Campus Location**	
Small regional campus	3 (14.3)
Large regional campus	2 (9.5)
Main campus	16 (76.2)

### Making sense of the change

The majority of participants were able to articulate a clear understanding of the motivation and rationale behind the change to an active learning curriculum. They cited several reasons, including the rapid change in the volume and accessibility of medical knowledge as well as changes in what students want from UME.

However, participants did convey they had some initial hesitations. These concerns frequently focused on what ushering in a new curriculum meant in reference to their *own* training. Some responses reflected classic generational “grumbling” that often accompanies any change, with one respondent explaining, “Clinicians definitely are…‘if it worked for us, then darn it, that’s what we’re going to do’” [125]. Another shared that some faculty “are very skeptical. They’re thinking, well, why change what I went through? It was good enough for me, why isn’t it good enough for the next generation of students?” [72].

The majority of responses about generational changes in UME, however, were quite reflective. For example, this participant articulated both reasons for and limitations of lecture-based medical education:

*Unfortunately, that was the way I was trained: regurgitation. I guess I overcame it. But I don’t think that’s the way modern students learn. […] And so, we’re having to learn how to not just teach memorizers, but people to solve problems and be good searchers, and know how to evaluate information for its quality, which, in the old days we didn’t do that.* [74]

As this next respondent candidly shared, the process of moving from skepticism to acceptance was self-referential as well:

*At first I was honestly a little skeptical.* […] *because I was trained in the prior era* […] *I thought oh, you know, here’s a fad in education. It’s a national fad. We’re just jumping on the train.* […] *Is this really a good idea or are we just trying to keep up with the Joneses? But as I saw it implemented and saw it roll out, I realized that it actually blended with my own philosophy of teaching a lot more than I had realized at first glance.* […] *it’s not a matter of providing content anymore, it’s a matter of enabling students and encouraging students to just engage their why—why do I need to know this. And so I realize that the ACE curriculum, that’s what it does.* [127]

This respondent, who referred to his own generation as “hunter-gatherers in the landscape of education” recognized that both the modalities and learning objectives in medical education had shifted since his training.

Even when faculty understood the rationale behind the curricular change and found it persuasive, it was hard to shed the influence of their prior training:

*I’m a product of passive education. Up until probably ACE I tended to be a lecturer rather than getting the students involved […] It’s difficult. I find myself going back and lecturing probably too much. Because as a product of that system and as someone that’s done that for many, many years, it’s tough to change ways in terms of going back and getting away from just saying ‘this is what’s important, this is what you need to know, put this in your brain and regurgitate at a later date.’* [72].

While a number of participants were still somewhat protective about their educational experiences, others had no problem criticizing their own training:

*I’m so jealous that I didn’t go through training like this. [in my training] […] you go in and you memorize, memorize, memorize, and then you take a test. […] Then when I got to the wards, […] I couldn’t take that knowledge and work back to the symptom and the clinical presentation. […] This way these students now are learning […] it is really turning it around. In everything they do, every activity they have, they’re already thinking like [a] doctor.* [97]

Overall, faculty made sense of the change by referencing their own training, and most participants shared a common understanding about the reasons behind the change.

### Grieving the Lecturer Educator Role

Once respondents found their own way to accept the curricular change, they were then confronted with the need to “unlearn” the way they had become accustomed to teaching. Commonly referred to as a shift from being a “sage on the stage” to “guide on the side,” [33] respondents shared candidly how they processed this shift. They often described an initial sense of grief that they were losing an educator role they loved.

*I think there’s a grief process.* […] *If you’re a longtime faculty here who’s, you know, been getting good [teaching evaluation] survey results and feel like your students are learning, and someone comes along and says ‘yeah, well that was great, but you’re going to do it differently now,’ even if they buy into it… someone just killed what you thought was the best and why you went into teaching. And even though you know that all good ideas eventually get a better idea, you want to hold onto the old idea. So, I think they have a grief.* [61]

Another participant echoed their personal difficulty with the transition:

*I went through my whatever, seven stages of denial and grief or something […] It was very difficult. I had a very hard time understanding what active learning meant and how to actually do that. I know that I have a good reputation as a fairly effective classroom speaker. I can lecture for hours on end and do a reasonably good job. Students like it. They give good, great evaluations. But converting that into where you’re making the students think was hard for me. It was really, really hard.* [128]

Faculty participants reported learning to re-think their previous teaching methods with a critical lens. One faculty member explained an experience of “giving up” their comfortable teaching approach:

*I think I’ve finally given up the ‘I know so much that it’s terribly important for me to vomit that all over my students.’ And I’ve found that they learn just as much, and I learn quite a bit. I’m happy with the paradigm.* [61]

In particular, respondents talked about the logistics of disassembling the centerpiece of the legacy curriculum: the lecture. Being forced to reassess material in lectures and take apart vetted, previously successful content was difficult:

*I had 15 years of content that I was sitting on, my absolutely phenomenally good lectures that I had to rip apart and throw in the trash and reconstruct. It was painful.* […] *very, very painful. So yeah, it’s not a straightforward thing.* [128]

*And I have cut more from my PowerPoints over the years than I ever added because I think one problem is—*[…] *if you love an area, you want to tell the students every interesting, exciting thing, and that can be far above and beyond what they need. And by doing that, then the main concepts get lost.* [124]

For educators like the faculty member above, their standard use of detail-dense lectures stemmed from a passion for their area of expertise. With the ACE curriculum’s focus on active learning, some of these beloved facts were no longer needed and were actually obstacles to facilitating critical thinking in the classroom. Even with these realizations, the change was painful, and faculty experienced grief, pain, and a sense of loss.

### Risking an active-learning educator role

While respondents described the process of grieving the old role and dismantling teaching styles and content, they also described learning how to embrace and enact the new role. Even for supportive faculty educators, they still had to learn how to be comfortable with the new active-learning teaching role, in which expertise was communicated differently. While taking off the former role was often described as causing grief, educators described embracing this new role as risky.

Some faculty explained that the feeling of risk for clinician educators came from a generalized discomfort physicians have with not being explicitly recognized as an expert, a feeling that being the previous ‘sage on the stage’ model had given them. One respondent explained that faculty in general are uncomfortable outside their content areas, and the integrative nature of ACE was pushing them outside those areas. A respondent shared: “I think they worry if they’re not content experts. There’s a risk for them” [89]. Another respondent described how leading a flipped classroom and flipping roles in the classroom felt risky:

*One of the contributing factors that make[s] [active-based teaching] uncomfortable for faculty is we are used to being the smart person in the middle of the room, and you know all the answers. And what flipped classroom does, or any kind of problem-solving teaching does, it reveals your own ignorance—a harsh word—but your own lack of knowledge or understanding about something.* […] *you may get to a place where you have to go, “You know, I don’t really know.”* […] *You become more on their level, someone who has to figure things out, too. You don’t just know—[snaps fingers]—the answer like that, if you do it right.* [79]

Being asked to join students “more on their level,” and learning to admit, even in front of a room full of students, that they did not know the answers to every possible question during an active learning session, was mentioned by multiple respondents.

*[Students are] giving you immediate feedback, and you kind of have to adapt and be a little bit more flexible, as opposed to just going in there, like, I’m going to give this lecture and you just plow through it. […] You don’t necessarily know what you’re going to get […] and it’s kind of fun. It’s also, I would imagine, for people who aren’t comfortable, very intimidating because you don’t know. You obviously have to get comfortable with saying “I don’t know,” or “that’s a great question, I’ll have to get back to you.” So, there are definitely some skills that you have to build. It’s a different way of*.

*engaging with students*. [68]

While this respondent enjoyed the “fun” of active learning, they acknowledged that many faculty would be uncomfortable with less control. Another respondent addressed this directly:

*I think one of the biggest challenges for most faculty is learning to shut up and not provide answers when a question is asked, […] to become comfortable with saying “I don’t know, that’s a great question, why don’t you look it up.” […] that’s the other thing that is a challenge that I think ACE is helping us address, and that’s getting people used to the idea that medicine is not an exact science. You’ve got to get real comfortable with uncertainty, with not knowing precisely what the outcomes of any given encounter is going to be.* [77]

In addition to getting comfortable not knowing everything in front of students, faculty also had to take the risk of admitting the need, in front of colleagues, to learn new skills for the new role:

*I think that faculty can be proud people, and they don’t like to ask basic questions in case they look foolish, particularly in a meeting, a faculty development meeting. So, they were forgetting that […] you’re not born with this knowledge. And […] if you haven’t picked it up so far, ask somebody*. [73]

Although faculty felt discomfort saying ‘I don’t know,’ admitting they needed new skills, and embracing a more integrative curriculum that stretched their boundaries, they ultimately progressed from grief to risk to identifying rewards.

### Identifying rewards of teaching in an active learning curriculum

While the process of changing roles was not easy, many respondents readily identified satisfaction with and rewards of their new role (see Table 2 for additional quotes).

**Table 2:** Participants’ quotes on the rewards of active learning-based teaching

Participant ID	Quote
61	“I’m happy with the paradigm.”
68	“For me, being able to see people actually engaged with your material as opposed to just sitting there listening is probably more fulfilling, in a way. But I also think the students are interesting.”
69	“I like this active learning modality much, much better.”
74	“…but it’s very enjoyable to see that the light’s going on and the brain’s clicking, and then actually coming up with the answers.”
77	“It’s been a very rewarding and very interesting dynamic to spend so much time with people who are just beginning to learn medicine.”
79	“I love this kind of teaching. That’s one thing I have no doubt about in my mind. This type of teaching is way more challenging and interesting for an educator.”
131	“…the collaboration, the teamwork, the spirited discussion, it’s been a lot of fun.”
72	“Oh, I think it’s more fun to engage the students in discussion, and to see lights go on in their head, and see them make connections.”
75	“Part of the fun of medical education is seeing the next generation of physicians and how eager and smart and service minded and dedicated they are, and finding ways to keep from turning them into cynics before they’re done. That gives me a lot of joy and energy. And so I get that much more in this curriculum than what we had before.”
81	“…we are on the forefront of medical education, and this is a really cool thing that we’re doing. I really like it.”
90	“…the teaching methods are kind of fun.”

Some faculty reported how much they enjoyed the more active discussion with students:

*I don’t know how fun it is to […] go through 60 or 100 slides on a PowerPoint presentation, are there any questions, and then leave the class. Now is that fun? Maybe it’s an ego trip to know that you’re the expert. But have I made an impact on the students? […] it’s much more fun when there’s an interaction between the students and the faculty member. And you can see the wheels clicking. You can see them digging down from something they learned earlier and say oh, that’s why we learned this.* [72]

This participant was able to disentangle the rewards of lecture-based teaching (i.e., “ego trip”) from the rewards of interaction and seeing learning happening. Witnessing moments of learning and comprehension was frequently reported:

*I’ve found that there’s ways of motivating that discussion and giving [students] just the right amount of additional guidance on where to look and how to look until somebody gets, you know, then they put it all together, like ah! It’s really gratifying to see that […] that switch turning. You see that almost every CBCL. If you’ve done it correctly, if you as the instructor have put together a [session] that’s well structured, you will be able to see that switch turn on in the small group.* [128]

Faculty also pointed out that this new way of teaching engaged them —as educators —more actively than the previous curriculum:

*And I’m a great lecturer, don’t get me wrong. I’m entertaining and witty and all that. […] But I would be lecturing in the legacy curriculum, and I was on auto pilot. I could be solving crossword puzzles in my head. There was really no intellectual engagement, or very little. […] Flipped classroom, I come out of there exhausted, like I really just sparred or something, because […] you don’t want to just tell them the answer, and so when they say something you’re trying to figure out why did they say that, and then what can I say to them […] to get them thinking about the right answer without revealing it, which is what you really want to do with every fiber of your being. And so that’s a real, […] intellectual challenge, and it’s fun*. [79]

For faculty, the active learning paradigm demanded they bring their full intellect and attention as educators. While taking more effort and work, faculty typically found it rewarding.

## Discussion

Our study examined the experiences of faculty educators during a substantial curricular change from lecture-based to active learning-based model at a state medical school. This paper focused on the process through which faculty navigated the change in their roles as educators. Overall, most faculty understood and supported the rationale behind the change, consistent with attitudes found in a prior study of faculty readiness at our institution [26].

Faculty often first processed the change reflexively, comparing it to their own training. Faculty described a sense of grief associated with leaving behind their familiar educator role, along with their favorite lectures. At a fundamental level, active learning flips not just the classroom but also the role of the educator. Faculty reported that learning how to enact the new active learning-based educator role felt risky, but ultimately yielded increased enjoyment from teaching, including greater engagement (from students and faculty) and satisfaction from seeing critical thinking and learning happening. Previous research has noted that faculty resistance to similar curricular change can stem from “a fear of losing power, control, and resources,” [9]and the faculty in our study confirmed the very real loss and grief from the changes brought by more active learning.

Our findings also suggest that studying the way faculty process and experience curricular change is an essential component of understanding and facilitating curricular change. Adapting to teach in an active learning-based curriculum challenges not only how one teaches but also the core goals of teaching. This finding is consistent with prior research showing differences in central beliefs about teaching between teaching-centered and learning-centered orientations [20, 21]. For faculty, grieving the loss of comfortable ways of teaching, breaking apart beloved lecture content, and learning to teach in a more interactive way were demanding intellectually and emotionally. Of note, prior research has shown a similar trajectory among excellent clinical teachers, who described moving from content experts to learner-centered facilitators as they progressed in their teaching experience [34]. However, in our study, the external stimulus for this shift (i.e. the curricular change) may have resulted in a greater sense of loss for participants than if the change resulted from purely intrinsic motivations. Within the model of professional identity formation that focuses on role, agency, and context as key forces for identity [10, 18], the curricular change can be viewed as a substantial change in the *context* for educators. Making sense of the change seems to be the part of the sensemaking process in which faculty tried to own the change themselves, bringing *agency* into alignment with context. Finally, as our findings showed, grieving an old, comfortable role and embracing a new role emphasize how connected *role* was for this sensemaking process.

The relationship between age and faculty’s experiences with role flipping found by others was not straightforward in this study [9]. A few respondents mentioned age as an impediment, especially for other faculty who continued to resist the change and stopped teaching completely. However, more respondents mentioned disconfirming cases, like younger faculty who were resistant to change and older faculty who welcomed it. While classic generational grumbling was present to an extent [35], our data suggest that time out of training and ease of adapting to the new curriculum did not have a linear relationship. This relationship could change in future years as new graduates trained under active learning-based curricula begin to fill teaching roles.

Significant curricular change requires intensive faculty development and training [25], and our findings also have implications in this realm for other schools implementing similar change. While creating new content and teaching materials was time intensive, it was also common for faculty to report challenges around learning how to manage uncertainty in the classroom and deal with the risk of not feeling like the expert. Faculty development could address these experiences directly and implement exercises (e.g., role playing) so faculty begin to learn to facilitate and practice scripts around limits to knowledge.

Our findings could also be helpful in understanding resistance to change that may emerge at different points in the change process. For example, faculty may conceptually agree with the curricular change but may resist it practically when forced to disassemble their lectures; others may falter at the role enactment stage, overwhelmed by the risk of being in front of a classroom without a clear roadmap for the conversation. Finally, some may experience frustration while being “flipped” and need encouragement and support along the way. For faculty in our study, experiencing the rewards of active learning-based teaching was helpful in solidifying beliefs that the hard work of change was worthwhile. Faculty development should focus on different needs, as others have suggested [36], educating faculty about these stages and designing specific tools and sessions for each potential bump in the road.

Our study had limitations. It was based on one institution, and while we believe the findings are likely transferable to other institutions, local contexts differ. Second, we sampled faculty who were involved in teaching before and after the transition to ACE in order to understand their process of change. We did not interview faculty who decided to step away from teaching in the ACE curriculum, and therefore our sample possibly captures a more positive view of the curricular change. However, our participants were quite candid about challenges and criticisms, and this combined with the researchers being located outside of the school’s office of medical education lowered concerns about social desirability bias impacting our findings. While our study focuses on the process of change for faculty who agreed to the change, future research could compare faculty who chose to change and those who refused.

In conclusion, our study shows that changing to an active learning-based curriculum in a medical school requires a considerable shift for faculty. We argue that faculty educators are themselves “flipped,” grieving the loss of their expert role and experiencing risk as they learn to facilitate active learning in the classroom. Research should continue to study the faculty process of change, and schools implementing change should adapt faculty development materials to match the stages of change faculty may encounter.

## Data Availability

The interview transcripts produced from this study are not publicly available because as part of the IRB-approved consent document, participants were informed that their data would not be shared outside of the research team. Additional de-identified quotes are available from the corresponding author on reasonable request.
